# A Quick Guide to Software Licensing for the Scientist-Programmer

**DOI:** 10.1371/journal.pcbi.1002598

**Published:** 2012-07-26

**Authors:** Andrew Morin, Jennifer Urban, Piotr Sliz

**Affiliations:** 1 Department of Biological Chemistry and Molecular Pharmacology, Harvard Medical School, Boston, Massachusetts, United States of America; 2 Samuelson Law, Technology & Public Policy Clinic, School of Law, University of California Berkeley, Berkeley, California, United States of America; Whitehead Institute, United States of America

Computing is ubiquitous in every domain of scientific research. Software is the means by which scientists harness the power of computers, and much scientific computing relies on software conceived and developed by other practicing researchers. The task of creating scientific software, however, does not end with the publication of computed results. Making the developed software available for inspection and use by other scientists is essential to reproducibility, peer-review, and the ability to build upon others' work [1], [2]. In fulfilling expectations to distribute and disseminate their software, scientist-programmers are required to be not only proficient scientists and coders, but also knowledgeable in legal strategies for licensing their software. Navigating the often complex legal landscape of software licensing can be overwhelming, even for sophisticated programmers. Institutional technology transfer offices (TTOs) exist to help address this need, but due to mismatches in expectations or specific domain knowledge, interactions between scientists and TTO staff can result in suboptimal outcomes.

As practitioners in the scientific computing and technology law fields, we have witnessed firsthand the confusion and difficulties associated with licensing scientifically generated software. SBGrid.org is a consortium of scientific software developers and users in hundreds of biomedical research laboratories worldwide. As facilitator and middleman between developers and end-users, we commonly assist in the dissemination and use of scientifically generated software. Through research and advocacy, the Samuelson Law, Technology and Public Policy Clinic works with software developers and other creators on licensing issues, particularly issues related to facilitating “open access” to scientific, technical, or creative materials. Together, we offer a primer on software licensing with a focus on the particular needs of the scientist software developer. The aim of this guide is to help scientists better engage with their institutional TTO when choosing software licenses.

## Why Software Licenses Are Important

Licenses are important tools for setting specific terms on which software may be used, modified, or distributed. Based on the copyright protection automatically granted to all original works, a software license—essentially, a set of formal permissions from the copyright holder—may include specific “conditions” of use, and are an important part of the legally binding contract between program author (or rights owner) and end-user.

Without a license agreement, software may be left in a state of legal uncertainty in which potential users may not know which limitations owners may want to enforce, and owners may leave themselves vulnerable to legal claims or have difficulty controlling how their work is used. This is equally true for software that is commercialized and offered for a fee, and software that is made available without cost to others. While end-users often balk at overly restrictive software licenses, the uncertainty caused when no license is given can also discourage those wishing to make use of a piece of code. It is important to note that licenses can be used to facilitate access to software as well as restrict it.

## Software Licensing in Academic and Research Environments

**Figure 1:**
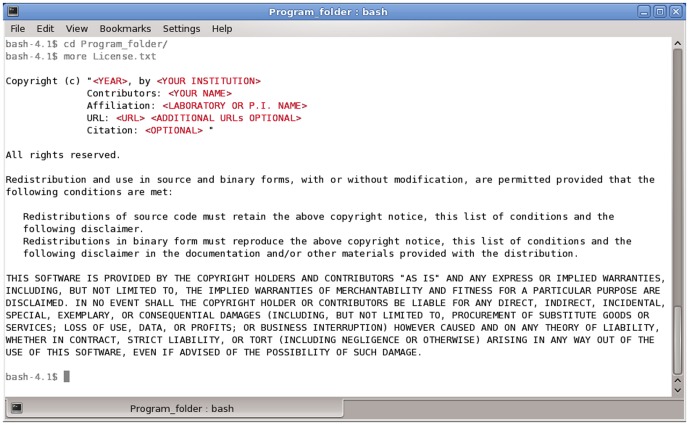
Example of FOSS license with “academic” style copyright statement. The example shown is the entirety of a 2-Clause BSD [8] license with copyright statement (at top, within quotes). The text of the license is in black. Red highlighted text is where the copyright holder applying the license inserts their specific information. Application of this and many FOSS licenses simply require that the text of the license be included (usually as “License.txt”) in the directory containing the distributed program binary and or source code.

For a license to be valid it must be granted by the owner of the work's intellectual property (IP) rights. Under the policies of most academic and research institutions, researchers who have created a piece of software are unlikely to own full rights to their works. Instead, the institution generally holds or shares legal right to developed software. Institutions' policies on IP ownership vary, but in most cases your institution will be the legal “rights owner,” and will be the entity that actually grants the license you choose for your software. Although many types of licenses, especially of the “free and open source” variety, are simple enough for the non-legal expert to understand and apply (Figure 1), it is generally necessary to consult your institutions' TTO before imposing a license. See below for more information about working with your institution in applying a license.

## Types of Software Licenses

Colloquially speaking, the spectrum of software licensing strategies can be divided into three categories: “proprietary,” “free and open source,” or a hybrid of the two.

### Proprietary Licensing

This strategy is familiar from the “click-thru” agreements that govern commercial software packages. The primary purpose of a proprietary software license is to limit the use of software according to the rights owner's business strategy. As a result, proprietary licenses are often very restrictive for end-users. They typically allow use of the software only for its stated purpose, often only on a single computer, forbid users from copying, redistributing, or altering the work, and specifically prohibit the creation of derivatives using parts of the work. Importantly, programs under proprietary licenses are typically distributed only in binary form and forbid examination of the program code or reverse engineering of any part of the program. In academic settings, proprietary software may occasionally release source code “for inspection purposes only” due to scientific publishing and peer-review requirements (Table 1).

**Table 1:** Summary of select attributes of cited licenses types.

	Name	Latest Version	Copyleft	Patent Grant^a^	Permits^b^ Code Linking	Used by^c^
**FOSS**	BSDMITECLApacheMPLLGPLGPL	2-Clause1.02.02.02.03.03.0	NoNoNoNoPartialWeakStrong	NoNoYesYesYesYesYes	YesYesYesYesYesYesNo	Gabedit, Chemkit, SciPyWeblogo, APBSRCrane, Sakai ProjectImagemagick, Autodock Vina, GenMAPPFirefox, ThunderbirdClustalW/X, IMP, BioJava, Taverna WorkbenchR Project, Perl, Coot, OpenBabel, GROMACS
**Proprietary**	Traditional “bespoke”^d^“Inspection only”^e^Commercial		NoNoNo	VariesVariesNo	VariesVariesNo	*Majority of scientist-created software* *Satisfies minimum publishing & peer-review requirement*MS Windows, iTunes, Acrobat
**Hybrid**	Any combination		Varies	Varies	Varies	Pymol, MySQL, BDB, Phenix

Note that the values assigned in the table are only a general summary of each license attribute and may not fully reflect the specific details of each license.

a License text explicitly describes the treatment of patents related to the software.

b Allows the linking of computer code under different licenses.

c Select examples of popular software employing these licenses.

d Refers to a range of custom-tailored licenses traditionally used by academic and research institutions.

e Traditional “bespoke” license that also makes source code available for inspection purposes only.

### Free and Open Source Software (FOSS) Licensing

Free and open source software (FOSS) represents a fundamentally different approach to software licensing. The primary intent of FOSS is to maximize openness and minimize barriers to software use, dissemination, and follow-on innovation. There are a wide variety of popular FOSS licenses [3], each of which vary in some important ways, but all grant free (as in freedom), open, and non-discriminatory access and rights to modify licensed software and associated source code. A common misconception is that FOSS is synonymous with “noncommercial.” In fact, as described by the two most influential definitions of FOSS [3], [4], “non-discriminatory” means that no category of user or distributor can be prohibited, including for-profit commercial entities. As such, FOSS-licensed software can be, and regularly is, commercially exploited. Some cited benefits of a FOSS strategy include widespread adoption, user contributions, and ease of collaboration [5]. Additionally, because of their open and non-discriminatory nature, FOSS licenses can simplify continued development and collaboration when researchers switch institutions, and when they collaborate across institutions. FOSS can also help to extend the useful lifetime of a piece of software beyond the direct involvement of the creators. We discuss some important differences in FOSS licenses below.

### Hybrid Software Licensing

Some software developers find that their needs are not well met by using either proprietary or FOSS licensing models exclusively. In these cases, “hybrid” (also called dual- or multi-licensing) approaches—combining a FOSS license with a proprietary “closed” license—are sometimes used. Under this strategy, the rights owner chooses which license to apply on a case-by-case basis. When ownership and licensing rights are clear, these licensing schemes can maintain some of the benefits of FOSS while also permitting creators to employ multiple business models [6]. The downside can be a significant added burden for the rights owner in applying, administering, and enforcing multiple licenses. This has generally limited the adoption of hybrid license models to large software development initiatives.

## Terms, Concepts, and Examples Useful in Understanding Software Licenses

### Open Source versus Closed Source

Source code is the human readable form of a computer programming language. “Open source” refers to licenses that require the source code be available to users, and that users be able to reuse, modify, and distribute the code [3]. Without access to source code, researchers cannot effectively inspect, understand, or manipulate the inner workings of a program. Source code availability is of increased importance in the context of scientific research, where peer review, reproducibility, and building upon prior work are integral to the advancement of science. Source code access helps researchers quickly identify and remedy bugs that might lead to spurious results and adapt programs or pieces of code to suit individual needs, and allows expert users to contribute to code development on an informal basis. An active open source user community participating in maintaining and improving the code base can free the original developer to concentrate on major enhancements or move on to other projects without sacrificing continued utility of the software.

### Permissive versus Copyleft

“Permissive” and “copyleft” are terms used to compare legal philosophies and attributes of FOSS licenses to traditional proprietary licenses.

*Permissive* licenses are those that place the fewest restrictions on users and adopters, often only requiring that the original creators be attributed in any distribution or derivative of the software or source code. For example, permissively licensed software may be incorporated into “closed” proprietary programs with no requirement that the source code be disclosed if the combined software is distributed. Permissive open source licenses are also sometimes called “research” or “academic” style licenses because of their origins in, and frequent use by, academic institutions [7].

Examples of popular permissive FOSS licenses include the Berkeley Software Distribution (BSD) [8], MIT [9], Apache [10], and Educational Community License (ECL) [11] licenses. The BSD and MIT licenses are often mentioned interchangeably due to very similar language and terms that accomplish largely identical goals. The primary intent of these licenses is to allow the use, distribution, and modification of your code for any purpose, while making sure that you as the creator receive credit for your work (see Figure 1 for an example of an FOSS license with an academic style attribution/citation copyright statement). The Apache and ECL licenses are similar in effect to the BSD/MIT, but include a license for patents related to the software (this can be desirable or not, depending on the situation—see below). The ECL differs from Apache in a slightly weakened patent grant to accommodate the often complex IP environments of academic institutions.

For developers who want to guarantee perpetual open source access to their work, some licenses employ the concept of *copyleft*, a punning reference to “copyright.” Copyleft uses copyright's legal framework to guarantee continued open access to a software and its source code. This is done by requiring, as a condition of the license, that any derivative works also be distributed under the same licensing terms as the original. These copyleft licensing terms are also sometimes referred to as reciprocity or “share-alike” provisions. Because of these reciprocity requirements, copyleft licenses are considered “restrictive” licenses, though these restrictions guarantee perpetual open access.

Examples of popular copyleft FOSS licenses include the GNU General Public License (GPL) [12], GNU Lesser General Public License (LGPL) [13], and the Mozilla Public License (MPL) [14]. The GNU Licenses are the most well known of all the FOSS licenses and have a strong community of supporters and advocates. Of these, the GPL has the strongest reciprocity requirements and is considered a “strong” copyleft license. The LGPL (the “Lesser GPL,” denoting its weaker copyleft requirements) is very similar to the GPL from which it is derived, but allows for linking to proprietary code under certain circumstances. Similarly, the MPL allows copyleft to be applied to some parts of the code and not others. The LGPL and MPL are considered a compromise between the strong copyleft of GPL and permissive licenses such as the BSD/MIT.

### Compatibility, Proliferation, Fragmentation, and Directionality

A fundamental goal of FOSS is to promote the free exchange of ideas and technology without fear of infringing the rights of others. Ideally, code licensed under like-minded FOSS terms should be freely combinable to create new products. *Compatibility* is the attribute of software licenses that allows combining of program code. To be compatible, license terms must be free of contradictory or mutually exclusive requirements. Alas, some FOSS licenses contain terms “incompatible” with other FOSS licenses, thereby diluting the ability to easily combine code.

This unfortunate situation has been exacerbated by the *proliferation* of incompatible FOSS licenses, many of which differ in only trivial ways. The Open Source Initiative (OSI) [15] was created in part to reduce the *fragmentation* of the FOSS license space cause by incompatible and redundant licenses. OSI thus strongly encourages using an existing FOSS license instead of creating a new, “bespoke” license, and offers a categorization of licenses to help developers avoid redundancy [16].

In general, the more restrictive the license, the less compatible it is with other licenses. Proprietary licensed software, by design, cannot be incorporated into other codebases absent a separately negotiated licensing agreement.

**Figure 2:**
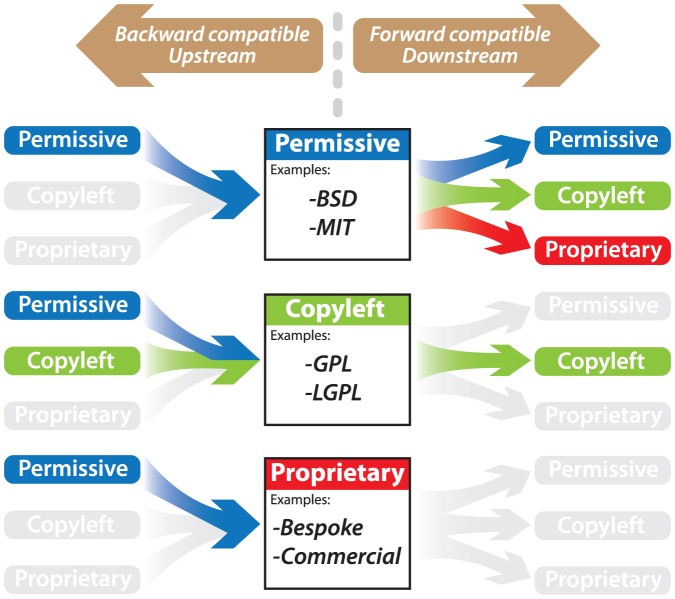
Schematic representation of license directionality. In general, permissively licensed code is forward compatible with any other license type. However, only permissive licenses, such as the BSD and MIT, can feed into other permissive licenses. Restrictive licenses like the GPL are backward compatible with themselves and permissive licenses, but must adopt the restrictive license from then on. Proprietary licenses can incorporate upstream permissively licensed code, but by definition are incompatible with any other downstream license. Grey represents actions that are not permitted without negotiating a separate license agreement with the rights owner.

License compatibility is further complicated, however, in that it is *directional*. License *directionality* refers to how a license behaves differently with code feeding into it (upstream, or backward-compatible) or out of it (downstream, or forward-compatible) (Figure 2). For example, a permissive license like the BSD is forward-compatible with nearly any other kind of license, but backward-compatible only with other permissive licenses. Likewise, a copyleft license like the GPL can incorporate (upstream) both permissive and other GPL'd code, but the resulting software may only be licensed (downstream) under the GPL.

Directionality is an important reason why, if you're trying to integrate code written by others with your own, you'll want to be aware of what license the code you are incorporating carries. When attempting to combine code from multiple projects each under different license types, issues of compatibility can become very complex.

### “Form” versus “Bespoke” Licenses

FOSS license are generally *form* licenses, meaning that their terms are standardized and a developer need only apply them (Figure 1). This standardization is critical to the success of FOSS strategies because it maximizes license compatibility and minimizes the cost of administering and understanding the terms of a given license. Conversely, *bespoke* licenses are custom-tailored for each individual project. Tailored licenses allow for greater control, but require more resources to develop and administer and are highly likely to be incompatible with other licensing schemes. Nearly all proprietary licenses are bespoke.

### Hybrid and Multi-Licensed Software

These license schemes differ from single licensing in allowing rights owners to choose which licenses best serve their needs on a case-by-case basis. One form of multi-licensing permits users and contributors to select among multiple licenses offered by the rights owner. Another example is when owners enter into separate “side” agreements not to enforce certain provisions of FOSS licenses, often for a fee. Limiting the reach of FOSS licenses in this manner is controversial within the open source community due to the partial circumvention of share-alike principles.

MySQL [17] and Oracle Berkeley DB [18] (BDB) are two well-known examples of multi-licensed software and are both made freely available for use, distribution, and modification under open source licenses. However, each of these programs is additionally offered for a fee under alternative licenses more amenable to proprietary business strategies.

## FOSS Licenses and Commercialization

It is a common misconception that FOSS licensing strategies preclude commercialization. In fact, OSI-approved [3] FOSS licenses cannot discriminate against commercial use. (This is one reason why institutional TTOs have sometimes preferred a bespoke “non-profit-use-only” license.) Though FOSS licenses preclude charging for the license rights themselves, developers are free to charge a fee for additional services such as technical support, priority feature development, consultation, etc. Hybrid licensing schemes (see above) offer further avenues for FOSS commercialization.

## Choosing a Software License

Determining which license will work best for you can require some thought, and depends not only on specific attributes of your software, but also on your particular goals. While both FOSS and proprietary licenses generally require attribution and include standard protections such as disclaimers of warranty, they differ in key aspects both philosophical and practical.

*If you want…*

…*the widest possible distribution and adoption, fewest restrictions on users, open and transparent source code, peer review, community contributions to the codebase, and easy incorporation of your code by others…* then a **permissive FOSS license** such as the BSD/MIT, Apache, or ECL licenses may work well. Because of the few requirements on users, these licenses are amongst the easiest to apply and administer, and promote unfettered incorporation of your code into other software—including copyleft or commercial software. Despite their general permissiveness, they do assure continued author attribution in any and all redistributions or derivative works.

…*to assure the benefits and openness of FOSS in all future derivatives of your work, open and transparent source code, peer review, community contributions to the codebase, and the potential incorporation of your code into other copyleft-licensed works…* then you should consider a **copyleft FOSS license** like the GPL, LGPL, or MPL. These licenses, by requiring anyone who distributes the unmodified or modified code to do so under the same license, guarantee perpetual open source of your work. Some copyleft licenses, such as the GPL, have particularly strong developer communities, encouraging community contributions to your software. The copyleft requirements of these licenses can sometimes, however, dissuade others from adopting or incorporating your code.

…*the ability to separately pursue proprietary models while leveraging the wide distribution, adoption, community contributions, and other benefits of open source software…* then a **hybrid or multi-license scheme** may be appropriate. Hybrid or multi-licensing can achieve the benefits of both open source and proprietary software licenses. However, as in everything, there is no free lunch. The legal, administrative, and organizational complexity of managing multiple licenses, as well as other administrative costs, often limits multi-license schemes to large software projects whose anticipated revenue stream justify the cost of dedicated licensing personnel. As noted above, this strategy is sometimes also controversial within FOSS developer communities.

…*protect the confidentiality of your source code, reserve maximum control over the distribution and use of your software, and derive licensing revenue…* then you should consider a **proprietary license**. Institutional TTOs sometimes default towards applying proprietary licenses due to staff's greater familiarity with them and a desire to preserve what is perceived (sometimes inaccurately) as the maximum potential for commercial exploitation. Institutions receiving public funds will typically license proprietary software to other academic or non-profit users at no charge but require a fee for licensing to for-profit and industry users.

## Applying a License to Your Software

Once you have chosen a license strategy for your software, the usual first step in applying it is to contact your institutional TTO. Although many FOSS licenses are easy to apply even by the non-legal-expert, as researchers and academics it is unlikely you personally own all of the rights to your work. Instead, these rights typically belong to, or are at least shared with, your institution. Therefore it is usually necessary to work with your institution when applying a license.

TTOs exist to help you make and execute these types of decisions. Nonetheless, coming with a clear idea of what kinds of licenses are available, which one you want, and why, will likely be both appreciated by your TTO staff and result in a more favorable outcome for you.

Once you've contacted your TTO, the process generally begins by helping the staff understand the “who, what, why, where, and how” of your work: how it works, who would be interested in it, what the innovation is, why you made it, where the funding came from, and other similar facts. Once TTO staff have this general understanding, they will discuss with you possible IP schemes—everything from placing the work in the public domain to creating a company to commercialize it. Most of the time, some form of license arrangement will be preferred. Be prepared, however. Some institutions' philosophies on protecting and exploiting IP are more aggressive than others. You may need to explain, for example, why using a FOSS license does not preclude commercialization (see above), why you think commercialization is not the most appropriate goal for your work, or why broad dissemination is an important goal for you. If you wish to propose a license that limits or forgoes the potential for generating revenue, you may first have to convince your TTO staff that your work lacks commercial value. While the process can sometimes be a bit of a negotiation, most institutions care a great deal about the scientific and societal impact of their IP, and we find that it is rare for an institution to act contrary to the express wishes of the creator of a work. Knowing what you want and why you want it should go far in making the licensing process as painless as possible.

## The Complication of Software Patents

An additional reason to contact your TTO before applying a license is software patents. Modern TTOs arose following the Bayh-Dole Act of 1980, which allows US research institutions to patent inventions developed using public funds and to license those patents [19], [20]. Because the vast majority of academic and research inventions are unlikely to have significant commercial value, most are never patented, but institutions typically require the disclosure of any patentable invention to the TTO. Many FOSS licenses (like the BSD or MIT licenses) are agnostic regarding patents, while some explicitly include patent grants in the license text (like the Apache or GPL licenses) (Table 1). Software patents are highly complex and generally outside the scope of this guide, but be aware that your TTO will want to discuss patent strategy, as well as copyright.

## Software Licensing and the Open Culture of Science

The needs and obligations of academic and publically funded research create unique considerations for scientist-programmers choosing a software license. Unlike in the software industry, where licensing strategy is primarily a matter of business strategy, it can be highly beneficial for scientists to publish, disseminate, and share the fruits of their work as widely as possible, independent of commercial potential. In addition, academic ethics encourage the wide sharing of research materials and information, including code. For programmers, this generally means sharing not just the binary executable, but also the source code so that others may use, validate, reproduce, and extend the work.

FOSS licenses such as those listed above are consistent with the open culture and obligations of scientific research, as well as the attribution and citation benefits academics have come to rely on. Permissive licenses may be preferred due to their ease of application and universal downstream compatibility. Copyleft licenses may be useful in accommodating upstream encumbered code or preferred by researchers seeking to assure perpetual open access, but their reciprocity requirements can limit downstream options. Hybrid licensing schemes, due to their added complexity, are more limited in their utility, but if appropriate, can offer many of the benefits of both proprietary and open source models.

Due to their closed and restrictive nature, proprietary software licensing schemes should probably be avoided whenever possible. As with other restrictive license models, the administrative burden of managing compliance and collecting revenues can be significant. For this reason, if anticipated total revenues are not high, it can often be more beneficial for scientists to take advantage of the reputational benefits and increased influence that come with the wide adoption and dissemination open licensing models encourage.

More broadly, especially in the context of scientific openness, collaboration, and peer review, the lack of available source code is a substantial drawback. In contrast to open source code, closed-source programs are essentially “black boxes” in the research workflow [21], opaque to both reviewers and users. The failure to release source code can be detrimental to the validation and acceptance of scientific results derived using the software. Although some traditional “bespoke” academic licenses attempt to mitigate the negative effects of proprietary licensing by offering software “free for non-profit use” or by publishing source code “for inspection only”, this nullifies the many significant benefits of community contribution, collaboration, and increased adoption that come with open source licensing.

**Editorial Comment**

**Andreas Prlić, Hilmar Lapp, Software Editors *PLoS Computational Biology***

Scientists are “dwarfs, standing on the shoulders of giants” (Bernard of Chartres). That is, in their pursuit to acquire new knowledge, they are building on the work of others. For this to be possible, already established scientific information must be widely accessible and reusable. This need for access to information is in conflict with a desire, the one to protect the value of intellectual innovation.

Copyright laws have been created with the goal of protecting the rights of copyright holders for a certain amount of time. In fact, in our software-dependent information age, few laws are influencing our professional (and personal) pursuits more than these. For example, at the time of writing this article, the two software giants Oracle and Google are facing each other in court over the question of whether Google's use of the Java programming language's application programming interface (API) infringed on Oracle's copyright. The outcome of the trial could have an impact on the freedom of software developers to use APIs and thus potentially hinder software interoperability.

Clearly, when developing software, choosing the terms under which the software can be reused, distributed, and built upon is an important consideration. Yet, many scientists and scientific developers have little training in or knowledge of the consequences of the choices they can make. Depending on how licenses are used they can either protect individuals' ability to capitalize on their creative works or ensure the public's ability to reuse. Licenses differ where in this spectrum they are positioned. This article, the “Quick Guide to Software Licensing for the Scientist-Programmer,” provides a summary of a variety of licenses and discusses their benefits and disadvantages. We hope that this guide helps in illuminating the seemingly complex jungle of licensing choices and their consequences, and that it serves as counsel to scientists and developers for what license is best suited in a particular situation.

*PLoS Computational Biology* supports open and unrestricted access to scientific publication and software. To foster a culture of open exchange and reuse of software, we have recently created a new category of Software Articles. For a manuscript to be published under this category in *PLoS Computational Biology*, we require that all software uses a license that is approved as open source by the Open Source Initiative (OSI). The approval criteria (http://www.opensource.org/docs/osd) set forth by OSI emphasize that the distribution terms must allow the software to be freely re-used, re-distributed, or modified. These requirements ensure transparency and reproducibility and, if applied to scientific software, push science forward by allowing researchers to build on existing work.
